# Modeling Human Brain Circuitry Using Pluripotent Stem Cell Platforms

**DOI:** 10.3389/fped.2019.00057

**Published:** 2019-03-05

**Authors:** Annalisa M. Hartlaub, Craig A. McElroy, Nathalie L. Maitre, Mark E. Hester

**Affiliations:** ^1^Center for Perinatal Research, The Research Institute at Nationwide Children's Hospital, Columbus, OH, United States; ^2^College of Pharmacy, The Ohio State University, Columbus, OH, United States

**Keywords:** human induced pluripotent stem cell (hiPSC), cerebral organoid, brain organoid, neurodevelopment, microfluidic, neural circuit

## Abstract

Neural circuits are the underlying functional units of the human brain that govern complex behavior and higher-order cognitive processes. Disruptions in neural circuit development have been implicated in the pathogenesis of multiple neurodevelopmental disorders such as autism spectrum disorder (ASD), attention deficit hyperactivity disorder (ADHD), and schizophrenia. Until recently, major efforts utilizing neurological disease modeling platforms based on human induced pluripotent stem cells (hiPSCs), investigated disease phenotypes primarily at the single cell level. However, recent advances in brain organoid systems, microfluidic devices, and advanced optical and electrical interfaces, now allow more complex hiPSC-based systems to model neuronal connectivity and investigate the specific brain circuitry implicated in neurodevelopmental disorders. Here we review emerging research advances in studying brain circuitry using *in vitro* and *in vivo* disease modeling platforms including microfluidic devices, enhanced functional recording interfaces, and brain organoid systems. Research efforts in these areas have already yielded critical insights into pathophysiological mechanisms and will continue to stimulate innovation in this promising area of translational research.

## Introduction

Neural circuits can be detected as early as 18–22 weeks gestational age (GA) during human brain development. Transient circuitries in the brain are initially established in the subplate, formed by afferent inputs from multiple brain regions, including the basal forebrain, brainstem, and somatosensory thalamus (1–4). Then through a process of synaptic refinement and dissipation of the subplate occurring between weeks 24 and 28 GA, more permanent connections are established within the cortical plate that include longer range thalamo-cortical and cortico-cortico circuits (1–4). During this early and vulnerable period, exposure to medications, drugs of abuse, or extrinsic factors such as maternal stress and infections has the potential to alter the establishment of neural circuitry. Post-natally, local circuits are fine-tuned and longer-range circuits such as cortico-striatal, meso-cortical, and cortico-hippocampal connections become increasingly organized and unified (4–7). The long-range neural circuits regulating motivation and reward, which have been implicated in drug addiction and mood disorders, are further refined at postnatal stages when neurons of the nucleus accumbens receive excitatory inputs from the prefrontal cortex (PFC), hippocampus, and amygdala as well as dopaminergic neuron inputs from the midbrain (8, 9). Neural circuitry dysfunction is also linked to neurodevelopmental disorders such as autism spectrum disorder (ASD) with deficits in cortico-cortico, cortico-striatal, and cortico-thalamic circuits (10, 11). In a mouse model of schizophrenia, genetic engineering of the human microdeletion 22q11.2 equivalent in mouse, resulted in alterations in functional connectivity between the hippocampus and the PFC (12–14). Modeling the specific brain circuits implicated in these disorders would allow for a basic understanding of circuit function and could provide mechanistic insight into how these circuits malfunction in the disease state.

Apart from understanding how neural circuits malfunction in disease, studying neural circuits also provides multiple benefits from a basic biological perspective. For example, with the development of sophisticated optogenetic approaches, it is possible to functionally map both short and long-range cortical connections at single neuron resolution within the mouse brain, or map microcircuit connections *in vitro* (15). In addition, understanding how neuronal circuits process information can be applied to the development of more advanced silicon-based electronic circuits. For example, by studying how neurons process information within the neocortex, an electronic circuit was conceptualized and designed that models a phenomenon called gain modulation, which is a non-linear method by which neurons process information from multiple sources (15).

Due to the experimental challenges associated with research on post-mortem human brain tissue and the inability of neuroimaging to provide cellular-level mechanistic insights, our current understanding of neural circuit function has largely been deduced from animal models. Recently, more physiologically relevant and tractable model systems based on human induced pluripotent stem cells (hiPSCs) are emerging to complement these animal models. Since hiPSCs were first generated (16–21), patient-specific stem cell lines have now been produced for a wide array of neurodevelopmental disorders such as ASD, schizophrenia, epilepsy, and ADHD (16, 17, 22–24). Lately, more complex brain organoid systems derived from hiPSCs have been developed, providing a more realistic three-dimensional model of human brain development with significant potential for modeling neural circuitry dysfunctions in neurodevelopmental disorders (25–33). In parallel, the development of microfluidic brain-on-a-chip devices that model defective neural circuits in disease may facilitate the investigation of pathogenic mechanisms (34–36). In this review, we discuss recent advances in the development of platforms using hiPSCs to model human brain circuitry, their advantages and challenges, as well as the use of microfluidic devices and other technological approaches to enhance their application in basic and translational research.

## Models for Reconstructing Human Brain Circuitry

The field of *in vitro* disease modeling has significantly accelerated following the generation of hiPSCs in 2007. The ability to harness patient-specific somatic cells and reprogram them into pluripotent stem cells indistinguishable from human embryonic stem cells (hESCs) has opened a novel area of research for modeling neurodevelopmental disorders. Development of robust hiPSC reprogramming methodologies has been an area of intense research with more than a dozen methods currently available (22). Although, the choice of reprogramming methodology ultimately depends on the research objectives, protocols that are rapid, low cost, and limit genomic integrations are the preferred methods of choice when deriving hiPSCs for disease modeling applications. A highly-efficient and RNA-based hiPSC reprogramming method utilizing primary neonatal fibroblasts recently achieved an 800% reprogramming efficiency, which is equivalent to 8 hiPSC colonies generated per fibroblast cell (37). Other methods allow simultaneous reprogramming and gene editing (to generate control isogenic hiPSC lines) from either fibroblasts or erythroblasts (38, 39). With improved methods of reprogramming and gene editing, hiPSC-based disease model platforms are increasingly being adopted.

Multiple hiPSC lines from patients with neurodevelopmental disorders have been generated that manifest similar functional deficits in neuronal cultures to those seen in the patients (31–33, 40, 41). For example, hiPSC-derived cortical neurons generated from patients with Rett syndrome display altered neuronal networks and synaptic deficiencies (42). Similar phenotypes have been described in cortical neurons generated from Fragile X syndrome patients containing mutations within the FMR1 gene (43). A more comprehensive summary of neuronal functional deficits generated from hiPSC-based neurological disease models can be found in several recent reviews (24, 44, 45). Alternative non-hiPSC sources such as the use of organotypic explant cultures or dissociated neuronal cultures to model three-dimensional neural circuits on a chip are not the focus of this review and have also been recently described elsewhere (46).Here we focus instead on the recent technological innovations in developing platforms to model specific brain circuits associated with neurological disease utilizing hiPSCs. We will also highlight opportunities to couple emerging technologies thereby increasing their utility in disease and disorder modeling (Table 1).

**Table 1:** hiPSC-based models to investigate neural circuit formation.

**Model type**	**Neuronal cell types**	**Description**	**Application**	**References**
Whole Brain Organoid	Glutamatergic (photoreceptors, retinal ganglion cells, bipolar cells, callosal neurons, corticofugal neurons); GABAergic and amacrine interneurons	Established proof of principle for neuronal connectivity and functional networks within brain organoids	Analysis of neuronal network dynamics	(30)
Fused Brain Organoid (Assembloids)	(i) Glutamatergic pyramidal neurons, GABAergic interneurons (ii) Glutamatergic pallium neurons, GABAergic subpallium interneurons	(i) Fused cerebral organoid model using GLU pyramidal neurons and GABA interneurons (ii) Fused spheroid model used to investigate Timothy syndrome	Local cortical circuitry (established through migratory GABAergic neurons) Local cortical circuitry (established through migratory GABAergic neurons)	(47) (48)
Microfluidic Chip	(i) Glutamatergic, GABAergic, dopaminergic (ii) Glutamatergic CA3 pyramidal and dentate gyrus neurons (iii) Medium spiny glutamatergic	(i) Fabrication of microfluidic device for analyzing neural circuitry (ii) Use of CA3 pyramidal and dentate gyrus neurons to explore perturbed hippocampal connectivity in schizophrenia (iii) Use of mouse neurons to model circuit disruptions in Huntington's disease	Three-way midbrain circuitry Mossy fiber hippocampal circuitry (DG-CA3 circuitry) Cortical-striatal circuitry	(34) (35) (36)^*^
Brain Organoid Transplant	See whole brain organoid	Integration, vascularization, and functional connectivity of human brain organoids into mouse brains	Cortico-cortico circuitry (*In vivo* model)	(49)

* *Used mouse primary neurons*.

## Brain Organoid Systems

Organoids are three-dimensional *in vitro* structures derived either from hiPSCs or adult stem cells (32, 40, 41). They are generated as a result of the ability of stem cells to self-organize and develop to form complex structures with organ-like features. Brain organoids, or “cerebral organoids” are hiPSC-derived tissue structures that recapitulate the morphological features and developmental processes of the developing human brain. Two main methods are utilized to generate brain organoids: (i) the un-directed or whole-brain organoid protocol that is based on the self-patterning ability of stem cells to develop with minimal extrinsic differentiation cues, or (ii) the directed differentiation protocol, which guides hiPSCs toward defined brain lineages using small molecules and extrinsic factors. The latter protocol can generate organoids with structures resembling the hippocampus, midbrain, cerebellum, and other brain regions. In contrast, the un-directed protocol generates brain organoids with greater cellular diversity and regional brain identities, but at the expense of increased inter-organoid variability. Drop-seq single-cell mRNA sequencing studies have demonstrated whole brain organoids contain considerable cellular diversity (Table 1). Six-month-old developed whole brain organoids were shown to contain cells predominately belonging to the neuroectodermal lineage such as astrocytes, oligodendrocyte precursor cells, neural progenitor cells, dopaminergic neurons, cortical neurons such as GABAergic and glutamatergic neurons, interneurons, retinal cells, and mesodermal progenitor cells (30).

Despite significant advances in our understanding of the cellular diversity and morphological complexity of brain organoids, only a few studies have investigated their electrical properties and neuronal connectivity. By performing extracellular recordings with high-density silicon electrodes implanted into 8-month-old brain organoids, researchers were able to identify spontaneous action potentials and coordinated bursting activity, suggesting that the brain organoids had established functional neuronal networks (30). Others have analyzed the electrical properties of neurons within brain organoid slices using whole-cell patch clamp techniques (29, 48, 50). Recent studies have demonstrated nested oscillatory network activity emerges within cortical organoids initiating at 6 months in culture, highlighting the potential to model the development of neural networks (51). Nonetheless, no study to date has investigated whether brain organoids can be coaxed to form local or long-range neural circuits.

Developing *in vitro* platforms for high resolution probing and manipulation of brain circuits remains challenging. New methods have generated regionally-specified brain organoids through the ability of two or more organoids to spontaneously fuse together. For example, several methods have been developed for generating dorsal and ventral forebrain organoids or spheroids (48) using parallel directed differentiation protocols followed by spontaneous fusion of the organoids or spheroids to study interneuron migration (47, 48, 52) and functional activity (48, 52). Fused regionally specified brain organoids, named “assembloids,” may reconstruct multiple types of brain circuits that have been implicated in neurological diseases, such as cortico-striatal, cortical-spinal, meso-cortical, and cortico-hippocampal circuits (33). This technology could model brain circuitry dysfunctions across a large number of neurodevelopmental disorders by combining the differentiation capacity of hiPSCs to generate diverse neuronal subtypes with their ability to produce arrays of assembloids.

Beyond *in vitro* studies, a new *in vivo* model of human brain neuronal connectivity was recently developed by transplanting human brain organoids into mouse brains (49). In this study, human brain organoids became vascularized, were integrated with microglia, extended long-range axons, demonstrated synchronized neural activity, and showed functional connectivity within the mouse brain. The ability of human brain organoids to integrate and generate functional neural circuits within the mouse brain opens up multiple opportunities for future exploration. One of the more compelling lines of research would be to investigate whether this transplantation method could restore neural circuit dysfunction in mouse models of neurodevelopmental disorders.

## Disease-on-a-Chip Microfluidic Platforms

The development of compartmentalized microfluidic devices has further enabled innovative methods to model human brain circuitry with an unmatched level of control. Microfluidic chip devices provide several novel features as platforms to model circuit-level connectivity *in vitro* such as: (i) the ability to physically segregate cell bodies of different neural subtypes while still allowing axonal growth between compartments (ii) compatibility with high-resolution video microscopy to investigate morphological and functional connectivity (iii) the ability to control axonal growth and monitor stages from early synaptic formation through late synaptic maturation. Thus, microfluidic chip platforms provide compelling systems to investigate neural circuit dysfunction in neurodevelopmental disorders.

As a proof of concept, a five-compartment microfluidic device was developed to model a mid-brain neural circuit composed of GABAergic, glutamatergic, and dopaminergic (DA) neurons (34). Using distinct fluorescent tags to label neuronal subtypes, allowed the visualization of glutamatergic axons projecting to a centralized compartment and forming synaptic connections with DA neurons. In addition, calcium imaging techniques demonstrated functional connectivity between neuronal compartments while whole-cell recordings and optogenetic stimulation techniques revealed circuit function.

In another study, a microfluidic chip platform modeled the hippocampal dentate gyrus (hDG)-Cornu Ammonis region 3 (CA3) circuit of schizophrenic patients (35). An efficient differentiation protocol was used to generate hDG and pyramidal human CA3 neuronal subtypes using hiPSCs from schizophrenic (SZ) and healthy patients (35, 53). Utilizing a two-compartment, microfluidic device connected by narrow channels, pre-synaptic hDGs were seeded into one compartment and post-synaptic hCA3s were seeded into the opposing compartment. A rabies virus tracing assay confirmed direct synaptic connections were established within this microfluidic system. Multi-electrode array (MEA) recordings and whole-cell patch clamp techniques established that the SZ hCA3 neurons had defects in both spontaneous and evoked electrophysiological activity.

A similar two-compartment microfluidic device reconstructed cortico-striatal circuits using primary neurons from a mouse model of Huntington's disease (HD). Though the main focus of this review is based on hiPSCs, this innovative system was included to emphasize the value of microfluidic systems in modeling brain circuitry. Data from this study suggested that presynaptic cortical dysfunction was both necessary and sufficient to induce pathogenic properties within post-synaptic striatal neurons, thus highlighting the critical value of these systems in providing mechanistic insights into disease pathogenesis (36). Through the combination of sophisticated optical tools such as glutamate and calcium reporter systems, HD cortical neurons demonstrated decreased glutamate release and overall reduced, but hypersynchronized, bursting episodes. An extension of this study should determine whether HD hiPSC derived cortical and striatal neurons show similar pathological neural connectivity deficits. Other neurodevelopmental disorders manifesting hyper-connected circuits such as ASD and ADHD could also be modeled using this microfluidic system.

As a potential extension of these studies and as a means to further understand the impact of multiple organ system interactions on human brain circuits, the linking of multiple organoid tissue types within a single microfluidic chip would provide for greater mechanistic understanding of neural circuit dynamics within a context of increased resemblance to *in vivo* conditions (54, 55). For example, recent studies have highlighted the complex signaling interactions between the gastrointestinal tract and the brain mediated through the gut microbiota (56, 57). To model these interactions, the development of an organ-on-a-chip platform that interlinks gastrointestinal organoids with an assembloid-derived brain circuit separated by an endothelial cell layer to mimic the blood-brain barrier would represent a further improvement in modeling accurate physiological responses on brain circuits.

## Current Challenges and Limitations

Despite the many advances, several challenges still exist to increase the physiological relevance of brain organoid systems and microfluidic platforms in modeling human brain circuitry. First, brain organoid cultures have variable and inconsistent internal morphological features, which limit their full potential. A recent study has shown that cellular diversity within brain organoids could vary within one-third of organoids of a given batch for a particular cell type such as forebrain neurons (30). Further, within an individual organoid, morphological structures such as ventricular zone-like and cortical plate-like structures can vary in size and organization. Recent improvements in bioreactor designs and organoid protocols have reduced this intra-organoid variability (29). Strategies that couple the use of microfluidics by precisely controlling media flow on brain organoids as they develop may minimize organoid variability. A recent study investigated whether a perfusable organ-on-a-chip system could be used to generate brain organoids (58). Although this novel system yielded organoids with enhanced neuronal organization, it is unknown whether this system could reduce organoid variability. Another source of variability stems from the use of different hiPSC lines, as they may contain significant genetic heterogeneity. In designing experimental studies, this issue can be circumvented by using isogenic or closely genetically matched hiPSC lines (59). Second, current protocols generate brain organoids that lack some of the brain cell types represented in the human brain such as microglia, endothelial cells, and oligodendrocytes, further decreasing their ability to model complex signaling events and neurodevelopmental processes. However, recent improvements to conventional protocols have yielded the generation of microglia within developing brain organoids (60), which are critical for the formation and maintenance of neural circuits (61). Thus, utilizing protocols that generate innate microglia within developing brain organoids would provide a model system with greater *in vivo* relevance to study neural circuits. Similarly, although current protocols generate brain organoids that lack functional blood vessels, it may be possible to bioengineer blood vessels within brain organoids or fuse them with blood vessel organoids based on recent advances in three-dimensional vascular organoid methods (62). Implementing this strategy may not only enhance nutrient diffusion throughout brain organoids, but would also provide relevant vascular cell types critical for maintaining neural circuit homeostasis through neurovascular signaling interactions (63–65).

Microfluidics approaches also have several intrinsic limitations in modeling human brain circuitry. Technical limitations include shear stress generated by microfluidic channels that can potentially harm cells or the microchannels can become easily trapped with cellular debris and air bubbles (66). In addition, the volumetric flow rate of microfluidic devices is considerably low throughput, thereby precluding the analysis of large sample volumes or assays that require rapid reaction kinetics such as exposing neurons to compounds that modulate their electrophysiological responses (66). In order to realize the full potential of organoid and microfluidics systems to model brain circuitry in neurological disease, future studies will need to overcome these obstacles.

## Future Directions and Applications

In the last decade, hiPSC research has continuously advanced the elucidation of neurological disease mechanisms and the search for potential treatments. More recently, advances in disease modeling techniques have led to the introduction of organoid platforms, which provide a more realistic model of organ development and have the potential to be more predictive drug screening platforms. In particular, brain organoids recapitulate many of the morphological features of the developing human brain. Despite significant advances in our understanding of the cellular diversity and morphological complexity of brain organoids, only a few studies have examined their electrical properties, neural oscillations or neural networks. With the development of new technologies to record extracellular field potentials from multiple neurons with high spatial and temporal resolution using multi-electrode arrays (MEAs), assays could be developed to record the electrophysiological properties of brain organoids in the context of normal development or exposure to environmental factors. Further, MEAs could be utilized as functional readout tools to phenotypically profile abnormal electrical activity arising from diseased brain organoids, thereby allowing for drug screening approaches to identify potential therapies that rescue electrophysiological deficits. Additional applications for MEAs include analysis of network topological properties and functional connectivity graphs within brain organoids based on spike train data, which could be derived using information-theory based network analysis (67–69). Additionally, the coupling of MEAs with optogenetics techniques would allow a greater level of analysis of neural circuit function by specifically controlling the activity of inhibitory or excitatory neuron subtypes. To further probe the function of neural circuits and also provide a phenotypic profiling tool in monitoring brain organoid neural activity, genetically encoded calcium or voltage indicators could also be integrated allowing for multi-level readouts of functional activity.

**Figure 1:**
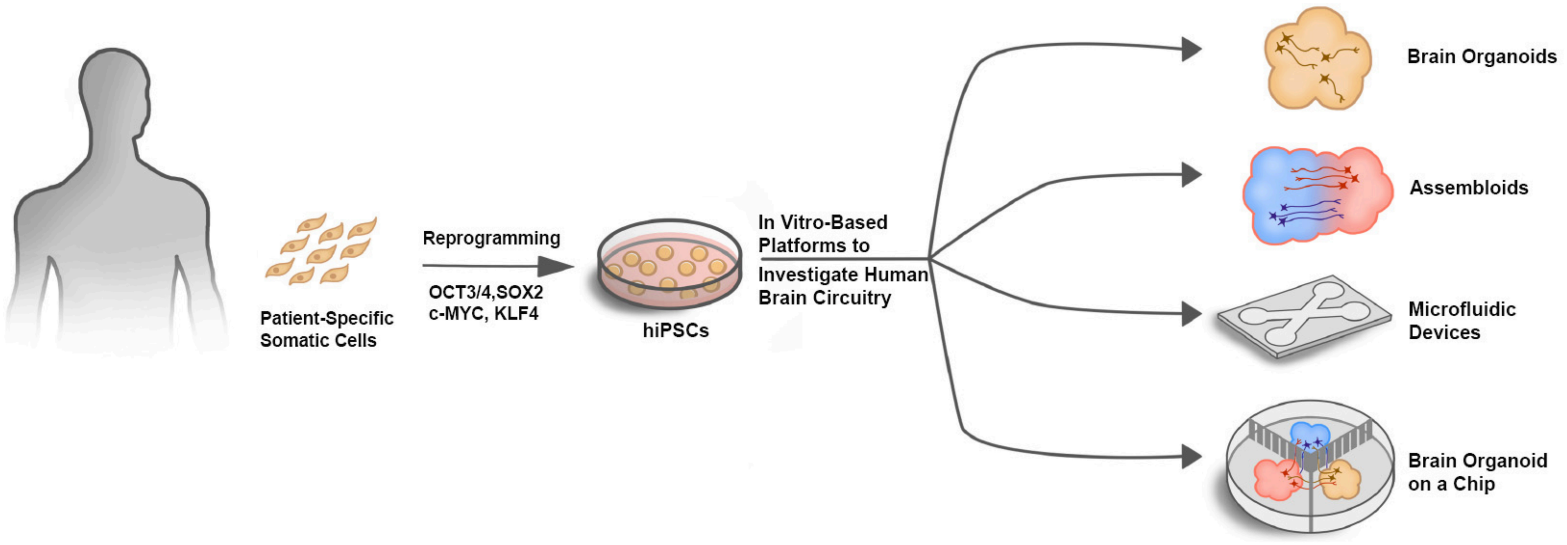
In vitro-based platforms to investigate human brain circuitry. Schematic illustrating different approaches to investigate human brain circuitry *in vitro*. Patient-specific somatic cells such as skin fibroblasts or peripheral blood mononuclear cells (PBMCs) that are easily isolated can be reprogrammed into hiPSCs using the Yamanka factors (OCT3/4, SOX2, c-MYC, and KLF4). hiPSCs can then be used as a source for generating multiple cell types in various platforms to investigate human brain circuitry.

In conjunction with neural circuit analysis within single brain organoids, regionally diverse brain organoids could be combined into assembloids to model long-range circuits such as those implicated in neurodevelopmental disorders and drug addiction. The same optical and electrical interface tools could then also be applied to these long-range circuit models. To further enhance the fidelity and reproducibility of assembloid-based platforms in modeling long-range circuits, microfluidic devices could segregate organoids into different compartments, while still allowing inter-compartmental projections and synaptic contacts between the separated organoids (Figure 1). Thus, combining the advantages of spatiotemporal control provided by a microfluidic device together with brain organoid cultures would allow a greater level of control and predictability in modeling brain circuitry dysfunction in neurodevelopmental disorders.

Finally, when considering the clinical translational potential of these *in vitro* platforms for modeling human brain circuitry, it is critical to remember the developmental context of many neurological disorders. Some neurodevelopmental disorders may be triggered early, potentially resulting from environmental exposures in combination with a susceptible genetic makeup; common examples are cases of parenteral heavy metal (e.g., mercury, lead, manganese) (70) or intrauterine drug (e.g., opioids, antiepileptics) exposures at key phases of neurodevelopment (71–78). Other disorders, such as cerebral palsy, start with a discrete lesion early in development and express varied developmental phenotypes, with neuroplasticity occurring in response to use-driven rewiring of circuits (79–81) or the introduction of neuroprotective agents (e.g., erythropoietin, caffeine) (82–84). New model systems accounting for the complexity of chemical, physiological, neuroelectrical, and genetic interactions with developmental timelines relevant to human processes will be invaluable in achieving true progress in clinically relevant neuroscience. Regardless of the many challenges that lie ahead for developing robust hiPSC-based models of brain circuitry, current systems have already provided key mechanistic insights into many neurological diseases, which is the essential first step toward developing efficacious therapies.

## Author Contributions

AH and MH conceived the main ideas and developed the structure of the paper. AH, MH, NM, and CM wrote the manuscript. AH prepared the figure and the table. All authors critically reviewed and approved the final manuscript for publication.

### Conflict of Interest Statement

The authors declare that the research was conducted in the absence of any commercial or financial relationships that could be construed as a potential conflict of interest. The handling Editor declared a past co-authorship with one of the authors MH.
